# The Governance of National Community Health Worker Programmes in Low- and Middle-Income Countries: An Empirically Based Framework of Governance Principles, Purposes and Tasks

**DOI:** 10.15171/ijhpm.2018.92

**Published:** 2018-09-18

**Authors:** Helen Schneider

**Affiliations:** School of Public Health and SAMRC/UWC Health Service to Systems Unit, University of the Western Cape, Cape Town, South Africa.

**Keywords:** Community Health Workers, Governance, Leadership, South Africa, LMIC

## Abstract

**Background:** National community health worker (CHW) programmes are increasingly regarded as an integral component of primary healthcare (PHC) in low- and middle-income countries (LMICs). At the interface of the formal health system and communities, CHW programmes evolve in context specific ways, with unique cadres and a variety of vertical and horizontal relationships. These programmes need to be appropriately governed if they are to succeed, yet there is little evidence or guidance on what this entails in practice. Based on empirical observations of South Africa’s community-based health sector and informed by theoretical insights on governance, this paper proposes a practical framework for the design and strengthening of CHW programme governance at scale.

**Methods:** Conceptually, the framework is based on multi-level governance thinking, that is, the distributed, negotiated and iterative nature of decision-making, and the rules, processes and relationships that support this in health systems. The specific purposes and tasks of CHW programme governance outlined in the framework draw from observations and published case study research on the formulation and early implementation of the Ward Based Outreach Team strategy in South Africa.

**Results:** The framework is presented as a set of principles and a matrix of 5 key governance purposes (or outputs). These purposes are: a negotiated fit between policy mandates and evidence, histories and strategies of community-based services; local organisational and accountability relationships that provide community-based actors with sufficient autonomy and power to act; aligned and integrated programme management systems; processes that enable system learning, adaptation and change; and sustained political support. These purposes are further elaborated into 17 specific tasks, distributed across levels of the health system (national, regional, and local).

**Conclusion:** In systematising the governance functions in CHW programmes, the paper seeks to shed light on how best to support and strengthen these functions at scale.

## Background

The last decade has seen a massive growth of interest in community health worker (CHW) programmes and their role in achieving health outcomes and universal health coverage.^1^ Emulating a number of well recognised programmes (in Brazil, Ethiopia, Nepal, and Iran, amongst several others), more and more low- and middle-income countries (LMICs) are seeking to develop national CHW programmes as part, or even the central element, of their primary healthcare (PHC) systems.^2,3^

National CHW programmes are not a new phenomenon. The current generation of reforms to community-based sectors is occurring on the back of longer histories,^4,5^ which have not only shaped attitudes, sometimes sceptical, towards national CHW programmes, but have also led to multiple, fragmented and uncoordinated initiatives on the ground.^6^ In many countries, new interventions in this sector are in effect a revitalisation, consolidation or expansion of existing forms of delivery that evolved over decades. These new initiatives have to take into account the varied and context-specific nature of existing community health systems if they are to be successfully implemented.^3^

In addition to their complex histories, CHW programmes face unique challenges, associated with their position between the formal health system, communities and households.^7^ Starting with the cadres themselves, there are questions relating to their roles, selection, identities, support and remuneration. The interface and relationships of CHWs with the formal PHC system, often described as precarious, need to be defined and actively managed.^8^ As roles expand and the significance of CHWs as frontline health providers grows, their ability to navigate the array of actors in community health systems also assumes increasing importance.^9^

As pointed out some time ago, CHW programmes are neither cheap nor simple, and the resources and systems capacity required to sustainably implement them at scale are considerable.^10^

National CHW initiatives thus need careful thought and attention to their governance, defined by the World Health Organization (WHO) as “overseeing and guiding the whole health system, private as well as public, in order to protect the public interest.”^11^ There is a growing conceptual and theoretical literature grappling with meanings of and approaches to health system governance,^12,13^ and recognition of the specific challenges and importance of CHW programme governance.^14,15^ A number of systems frameworks for CHW programmes have been proposed that incorporate notions of governance^16,17^ but with a few exceptions,^8,18^ there is little in the way of empirical evidence or guidance on the everyday practice of CHW programme governance, and what is required across levels of the health system to successfully implement programmes at scale.

Drawing on a combination of empirical observations of reforms to South Africa’s community-based health sector, conducted by the author, and conceptual thinking on health system governance, this paper proposes a multi-level framework for the governance of national CHW programmes in LMICs, particularly relevant to federal or decentralised health systems.

The framework is presented as a set of principles and a matrix of 5 key purposes and 17 tasks distributed across levels of the health system, with a focus on the *practice* of governance. The framework can be regarded as both descriptive and normative, although its emphasis remains on “how things are done,” rather than an idealised and abstract list of “what should be done.”^19^ Its aim is to systematise and organise the tasks of CHW programme governance as they present themselves to the stewards of these programmes, most often public sector managers, in order to:

- Provide a better appreciation of CHW programme governance functions across levels of the health system, from national, to regional and local;
- Establish the range of governance tasks and capacities required of CHW programmes;
- Support prospective monitoring and action on CHW programme governance.

The paper begins by describing the inputs to the development of the framework (ie, the methodology). It then presents the framework itself, its underlying assumptions and principles, purposes and tasks, and the associated capacity requirements. The paper concludes with a discussion of the framework in relation to CHW programme experiences elsewhere, its limitations, applicability across diverse contexts, and potential uses.

## Methods: Constructing the Framework

The framework was constructed from both empirical and theoretical inputs, with each set of inputs contributing assumptions, principles and specific tasks (Table 1).

**Table 1:** Inputs Into the Multi-level CHW Programme Governance Framework

**Specific**	**Contribution to Assumptions and Principles**	**Contribution to Framework Design and Tasks**
**Empirical Observations From the South African Experience**		
Case studies and cross case analysis of provincial implementation Participant observer in national policy processes	Programmes are path dependent and show sub-national variation Governance as distributed and negotiated Importance of local relationships The practice of governance is multifaceted combining analytic, technical, managerial and political roles	Provides the overall structure and content of the framework
**Conceptual/Theoretical Inputs**		
Sub-functions approaches	Importance of fundamental values (eg, participation, transparency)	Framing of specific tasks: Direction/policy Programme structure and systems design Information/intelligence Partnerships Inter-sectoral action Systems of accountability
Polycentric, state-society approaches	Decision-making and power distributed between state and non-state and community players	Network/collaborative/horizontal relationships
Multi-level governance	Governance (and power) as distributed, vertically and horizontally Implementation as non-linear, iterative, and negotiated	Governance as a negotiated process of co-production at all levels Programme design as bottom-up/top-down requiring systems of feedback and adaptive learning

Abbreviation: CHW, community health worker.

### Empirical Inputs

The primary, empirical inputs into the framework were a series of case studies, conducted by the author and colleagues over 2 years (2012/2013), of South Africa’s Ward Based Primary Health Care Outreach Team (WBOT) strategy.^20-22^ The WBOT strategy is South Africa’s version of a national comprehensive CHW programme, which built upon a pre-existing non-governmental organisation (NGO)-based community care and support system that emerged as a response to HIV/AIDS in South Africa. The strategy was first introduced in 2011 and has since been implemented, with varying degrees of success, across the 9 provinces of the country.^8^ It was recently formalised in a policy statement.^23^

Case studies were conducted of the adoption and initial implementation of the WBOT strategy in 3 provinces – the economic heartland of Gauteng, the urban-rural mix of the Western Cape and rural North West province. These case studies were multi-method in approach combining collation of documentary evidence (including routine health service data), interviews (individually and in groups, open-ended and structured) with a range of players, and observations of practice. The case studies were conducted by researchers working in the different provinces, but designed jointly by a network of national collaborators, led by the author. A total of 146 individual and 20 group interviews were conducted in these case studies, although the scope and depth of each study varied (for a summary of data collection in each case study see Schneider and Nxumalo^8^).

The 3 case studies were written up as individual reports.^20-22^ These reports documented the context specific ways in which the WBOT strategy emerged in South Africa at sub-national level, as a negotiated product of provincial and local histories of community-based services and the new mandates from the top. Collectively the findings surfaced the key issues and challenges related to the reorientation of community-based and PHC services, and the new systems, relationships, resources and sub-national buy-in required.

Following the individual case studies, an inductive cross case analysis (detailed in Schneider and Nxumalo^8^) specifically sought to frame and characterise these challenges as a set of sub-national CHW programme governance and leadership tasks. These were synthesised into 4 key roles: (1) negotiating a fit between national mandates and provincial histories and strategies of community-based services; (2) defining organisational and accountability relationships between CHWs, local health services, communities and other players; (3) developing aligned and integrated management systems; and (4) leading change processes and maintaining political support.

This cross case analysis highlighted the multi-faceted and distributed nature of governance roles, the mix of hierarchical, collaborative and contractual governance relationships involved, and the capacities these entailed.It also illuminated the context specific and path dependent nature of CHW programmes, pointing to the futility of detailed, universalist blue-prints and the importance of feedback loops and bottom-up/top-down dialogue in such programmes.^24^

Added to these formal evaluations were the observations and reflections of the author from participation in national policy processes in South Africa, first as part of civil society consultations, and then in a Ministerial Task Team, which developed the WBOTs strategy in 2010/2011.^25^ This was followed by technical support to the development of national monitoring and evaluation (M&E) systems for the WBOTs (2012/2013). Through this national involvement it was possible to reflect on appropriate roles of the centre and periphery in decentralised CHW programme governance. A specific observation over this period was of insufficient national political, budgetary and policy commitment to WBOTs and the constraints this posed to implementation at sub-national level. This observation was put forward and validated in a presentation on the state of the WBOT Strategy at a national conference.^26^ Mobilising and sustaining political support was thus identified as an additional fifth role to the 4 sub-national governance and leadership roles identified in the provincial cross case analysis, where political commitment had been subsumed into 1 of the other roles.

Table 2 summarises the key contributions of each source of data to the framework.

**Table 2:** Summary of Empirical Inputs Into Framework

**Source**	**Key Findings and Contributions**
Provincial case study 1: North West^20^	• Early adopter and successful implementer of WBOT Strategy • Governance as managing change, including the importance of common collective visions, participatory planning and feedback mechanisms • Technical system (human resource, finance etc) reorganisations required for implementation • Community dialogues as key to implementation • The complexity of relationships between facility and community-based teams
Provincial case study 2: Western Cape^21^	• Well established pre-existing provincial model, with organisational resistance to new strategies • Reorientations in technical systems and local organisational relationships required to shift from disease specific to comprehensive approaches integrated into the PHC system • The importance of shifting managerial mind sets and styles in the PHC and district system from command and control to the more collaborative, horizontal approaches required to engage other players in community health systems • The role of priority setting and planning at sub-district level
Provincial case study 3: Gauteng^22^	• Traditions of innovative district-level community-based programmes having to negotiate the “fit” with the new national strategies • The value of independent physical locations (“health posts”) in enabling the autonomy of community-based teams from facility level demands • The mechanisms of community level inter-sectoral coordination
Cross case analysis^8^	• Governance and leadership tasks in the above case studies synthesised into four key roles
National observations^26^	• Insufficient national political commitment identified as a key constraint to sustainable implementation at provincial and district level • Delays in finalising policy (expressed commitment) • Weak national planning and coordination mechanisms (institutional commitment) • Lack of ring fenced resources for expanded community-based services (budgetary commitment) • Little priority given to information and research on WBOTs

Abbreviations: WBOTs, Ward Based Primary Health Care Outreach Teams; PHC, primary healthcare.

Based on the sub-national case studies and national observations, the governance roles of CHW programmes were thus grouped into 5 categories and form the overall structure of the matrix and its bottom-up nature. These roles are: (1) negotiating the policy to be adopted, (2) reconfiguring frontline relationships, (3) technical systems development, (4) leadership of change, and (5) generating and sustaining political support. The specific tasks linked to each role emerged principally from the case studies but were also informed in their framings by the conceptual literature on governance and the empirical literature on CHW programmes.

### Theoretical Inputs

The literature on health system governance provides conceptual and technical inputs to the framework, serving to clarify assumptions, establish principles and frame specific tasks. Although relatively small, the governance literature encompasses a wide range of approaches, definitions and key assumptions.^13,27^ For the purposes of conceptualising national CHW programmes, it is categorised into 3 broad approaches. Firstly, the “*sub-functions*” approach to governance, outlined in the WHO’s building blocks framework on health systems,^11^ and subsequent adaptations of this approach.^27-29^ This literature spells out specific components of health sector governance (such as policy, regulation, and accountability) while also offering principles of good governance (such as participation and transparency). It is strongly oriented towards ministries of health and the design aspects of governance.

Secondly,* polycentric* approaches, bringing into focus governance actors other than the state. Brinkerhoff and Bossert^30^ propose 3 key players in health system governance relationships: the state, providers and citizens/clients, defining governance as “…the rules that distribute authorities, roles and responsibilities among societal actors and that shape the … interactions among them.” This wider state-society view of governance is especially relevant to CHW programmes, which typically involve an array of governmental, non-governmental and community actors in governance relationships.^6^ The distribution of roles, authority and power between these actors is thus a key consideration in CHW programme design and implementation.

Thirdly,* multi-level* approaches that view governance as distributed, not just horizontally between societal actors, but also vertically across government levels. This is particularly the case in systems with a degree of decentralised decision-making. Multi-level governance involves iterative processes of *negotiation* and co-production across the health system, in which there is “sharing [of] responsibilities and power of influence, both horizontally (between ministries and between actors at a local level), and vertically (between various government levels).”^31^

Multi-level approaches also view governance as encompassing all phases of the policy process, including implementation. In Hill and Hupe’s “Multiple Governance Framework,”^32^ actors engage in 3 forms of governance:

- Constitutive governance: “the fundamental decisions about the content of policy and about the organisational arrangements for its delivery”
- Directive governance: “facilitating the conditions for the realisation of collectively desired outcomes”
- Operational governance: “managing the realisation process”

These forms of governance are not synonymous with administrative levels, and all forms of decision-making may be present at any level. For example, where the state has a weak stewardship role in CHW programmes, the interactions between local players (community, NGO, providers) may define the fundamental rules of the programme. Conversely, national ministries may be directly involved in operational governance (such as training).

## Framework Assumptions, Principles and Content

### Governance Assumptions and Principles

Drawing on the empirical and conceptual inputs discussed above, the framework assumes, firstly, that national CHW programmes are complex initiatives designed and implemented in complex adaptive systems; and secondly, that reforms to national CHW programmes build on longer histories of community-based action and have to take this into account, balancing central direction with the sub-national and local adaptation required for adoption and assimilation.

With respect to governance:

- CHW programme governance is defined as the rules that distribute authorities, roles and responsibilities among societal actors and that shape the interactions among them.^30^
- Governance emerges from negotiated and deliberative processes across multiple interfaces, and has both hierarchical (vertical) and network or collaborative (horizontal) components; it is distributed across levels of government (multilevel) and amongst a range of state and non-state actors, including non-governmental, community, provider and international actors (polycentric); the core “rules” of CHW programmes are expressed in the relationships amongst these actors;
- Governance straddles all phases of the policy process (agenda setting, design, implementation and evaluation), which unfolds in a non-linear and iterative fashion; governance values such as participation and transparency are key to learning and adaptation in the policy process;
- CHW programme governance entails more than the technical design of regulatory and oversight mechanisms, structures and plans (hardware), it also requires the ability to manage a complex array of non-hierarchical relationships, and the strategic and tactical ability to steer implementation and to recognise and make use of political windows of opportunity (software);
- The state is the main steward of national CHW programmes, most commonly implemented through public health systems, although the degree of direct government involvement in governance relationships will vary across contexts;
- The capacity for CHW programme governance encompasses not just the competencies of specific governance actors, but also the systems of support for these actors, the extent of collaborative networks between system actors and the broader institutional environment in support of the values and principles outlined above.^33^

### Matrix of Purposes and Tasks and Associated Capacity

The matrix is organised into 5 key CHW programme governance purposes, based on the empirical case analysis, and framed as intermediate governance outputs. They are:

1. Policy mandates as a negotiated fit between evidence, histories and strategies of community-based services
2. Defined organisational and accountability relationships between CHWs, local health services, communities and NGOs
3. Aligned and integrated planning, human resource, information and financing systems
4. Leadership of change, and capacity for ongoing learning and adaptation
5. Mobilised and sustained political support

These purposes are further elaborated into 17 specific tasks, presented in Table 3.

**Table 3:** A Multilevel Matrix of National CHW Programme Governance Purposes and Tasks

**Governance Purposes (Outputs)**	**Specific Tasks**		**National**	**Provincial/** **Regional**	**District/** **Local**
Policy mandates as a negotiated fit between evidence, histories and strategies of community-based services	1	Policy as a negotiated fit between international evidence, national policy and provincial/regional and district/local history, norms and practices in CHW programme roles and design;			
	2	Assessment of strengths and weaknesses of current system, organisational readiness for change and public acceptability;			
	3	Established programme ownership, identity, structures of oversight and coordination (vertical and horizontal) relevant to the context;			
Defined organisational and accountability relationships between CHWs, local health services, communities and NGOs	4	Defined roles of CHWs, NGOs, communities, formal providers and other actors of the PHC system, ensuring community-based services retain the autonomy and power to act;			
	5	Mechanisms of collaboration and coordination with other actors in the community health system, and support for inter-sectoral action on the local social determinants of health;			
	6	Systems for performance, financial and community accountability;			
Aligned and integrated planning, human resource, information and financing systems	7	Local capacity for priority setting, planning, contracting and/or coordination;			
	8	Fair CHW remuneration and incentive-based systems;			
	9	Supportive supervision, referral and communication systems through the PHC system;			
	10	Accredited basic and in-service training aligned to roles;			
	11	An effective M&E system;			
Leadership of change, capacity for ongoing learning and adaptation	12	A vision that is collectively owned;			
	13	Clear change management and organisational learning strategies with frontline participation and feedback mechanisms based on transparency and sharing of information;			
	14	Meaningful partnerships to support change processes and ongoing renewal;			
Mobilised and sustained political support	15	Promotion of well evaluated local experiments and avenues for evidence to be communicated; modelled costs of the strategy against the benefits to be gained;			
	16	Political windows of opportunity exploited;			
	17	Budgetary commitment.			

Abbreviations; CHW, community health worker; NGOs, non-governmental organisations; PHC, primary healthcare; M&E, monitoring and evaluation. Note: shading denotes degree of relevance for that level based on the South African case: the darker the greater the relevance.

The first governance purpose, policy mandates as a negotiated fit between evidence, histories and strategies of community-based services, is concerned with the design of CHW programmes. It entails a process that looks both backwards and forwards – backwards in finding the fit between international evidence and prior national and sub-national histories, needs and strategies, and forwards in assessing organisational capacity, actor readiness and public acceptance for change. It is also presented as a negotiated process of accommodation between actors at different levels of the system, where top-down mandates can meet bottom-up innovation and problem solving. This is not a once-off event and likely to emerge iteratively over time.

Through this process, a more fundamental set of questions regarding CHW programme identity and ownership are settled, such as:

- Should CHWs be conceptualised as agents of community mobilisation or as an extension of the health system?
- Should they become civil servants and considered as part of the health workforce or be managed through non-governmental intermediaries?
- Are CHW programmes to be presented principally as implementers of a core package of technical disease interventions or do they have a broader household and community role?
- Who are workers accountable to and who do they identify with – communities or the health system?^10^

The capacity requirements for this governance function are an analytic ability to marry global evidence with local contexts, drawing on formal as well as tacit, social and situational knowledge to determine what works for the particular setting. It requires recognising CHW programmes as having unique trajectories and the importance of resisting top down or imported models which may be favoured by donors or partners. It may also require the political ability to manage resistance from other players in the health system and the technical capacity to address regulatory or legislative barriers to new roles.

The second purpose addresses the (re)-configuration of local organisational and accountability relationships, identified as a key challenge in the case studies. It highlights the importance of programme structures that balance coordination and integration with the formal primary healthcare system with some degree of autonomy on the part of CHWs. This is necessary to ensure that CHWs do not become lowly players at the bottom of a health worker hierarchy, drawn into health facilities as an “extra pair of hands.”

Following Brinkerhoff’s typology,^34^ 3 components of accountability are proposed for CHW programmes: performance, financial and community. They combine mechanisms of vertical accountability through hierarchies with horizontal accountability between players in the community health system, based on responsiveness and collaboration.

The capacity requirements for this second purpose include organisational design, the technical development of management systems and the ability to engage and navigate the formal and informal worlds of both hierarchical and collaborative governance relationships.

The third purpose involves the development of new systems – planning, priority setting, human resource (including training and supervision), M&E, and referral. These systems need to be aligned with each other and also integrated into the health system. The governance tasks in this purpose entail, amongst others, mobilising specialist technical expertise in the design and implementation of core management systems, broader systems knowledge to facilitate integration with other systems, and the ability to generate support, internally from the health system and externally with partners.

The introduction or strengthening of CHW programmes has ramifications for many other elements of, and players in, the health system. The fourth purpose focuses on the leadership of change, a strategic role that requires building momentum and constituencies for change, developing implementation plans and strategies (such as pilots), mobilising and aligning teams, and instituting systems of monitoring and feedback. This domain requires the ability to generate and communicate shared visions, draw the attention of and mobilise actors around this vision, and to design systems of participation, feedback and learning.

The final purpose, a key challenge identified in the South African setting, is mobilising and sustaining political and budgetary support for CHW programmes. This is enabled by the generation of evidence from well-evaluated experiments in real-life local settings, monitoring impacts at scale, and the development of “investment cases” (outlining costs and benefits).^35^ It requires a capacity to manoeuvre politically, including around professional interests and resistance, to recognise and make use of windows of opportunity and communicate messages, draw in technical expertise when needed and embed research within the national strategy.

While the specific tasks are presented as discrete elements in the 5 categories, there are overlaps and connections between them. For example, the analytic focus of the first 2 purposes link to the strategic management roles of developing shared visions further down in the matrix; managerial systems of accountability and sharing of information link closely to the technical processes of designing effective M&E systems and establishing costs and benefits with ensuring fair remuneration.

All functions are distributed across levels (national, regional, local) of the health system, recognising the contingent, context-specific and evolving nature of needs and strategies, and avoiding one-size-fits-all approaches. Based on the South African experience, the matrix suggests different emphases (denoted by degree of shading) in the distribution of roles across spheres. Priority setting and planning is emphasised as a district level function, while aspects such as political and resource mobilisation are primarily roles of the centre (national sphere). Policy is presented as the outcome of negotiation between international evidence, and regional and local history and practice. As they often need to engage national regulatory processes, employment regimes and the design of accredited training programmes are proposed as a central function. In other contexts, specific levels within government and the distribution of roles between them (ie, the shading) would be different.

## Discussion

The governance of national CHW programmes can be understood as a distributed function, negotiated across levels of the health system and as encompassing analytic, managerial, technical and political roles and capacities. Successful governance includes being attentive to the prior history of community-based initiatives when implementing new strategies; enabling equity of voice and power at a local level; focusing on the technical elements of programmes, whilst also shaping their values, orientations and visions; and mobilising alliances, resources and political support. These processes embody more than the technical design of structures and policy, conducted as planning exercises by experts behind closed doors. They are dynamic, iterative and negotiated processes across multiple interfaces, requiring “learning-by-doing” and political acumen. The framework presented in this paper seeks to make “actionable” these understandings of CHW programme governance.

In the absence of other literature or guidance taking a holistic perspective on the governance of CHW programmes, it is not possible to assess the validity of the framework and its constructs. However, elements of the framework resonate with, and have been shaped by, empirical findings on CHW programmes elsewhere.

In their multi-country assessment of the implementation of integrated community case management (iCCM) of childhood illness in 6 African countries, Bennett et al^3^ highlighted alignment with national contexts as key to the uptake of iCCM strategy. The most recognised national CHW initiatives, such as the Ethiopian Health Extension Worker Programme, the Indian Accredited Social Health Activists (ASHAs), or the Brazilian Family Health Teams, all have unique cadres, characteristics and identities.^16^ These have emerged in highly context specific ways, responding to particular political, health system and other imperatives. This observation accords with the design of, or reforms to national CHW programmes as “negotiating fit” between a variety of context specific or situational factors and a growing global evidence-base on role and practices of CHW programmes.

Much has been written on the complexities of the interface between CHWs and other players in the PHC system. Across contexts, CHWs complain of being under recognised, and poorly respected and supported by formal health sector players.^36-38^ CHWs risk being drawn into health facilities as menial workers, especially if they are answerable to facility-based providers.^39^ Unless these unequal power relationships and divergent interests are addressed, CHW programmes may fail.^4,5^ There are a number of ways in which CHW programmes have managed frontline relationships to protect the autonomy of community-based teams. One approach, almost counter-intuitively, has been to separate the management and employment/payment of CHWs from the rest of the PHC system, as happened in the inception phases of the Brazilian Family Health Programme^40^ and which is currently the case in Malawi^41^ and Chhattisgarh State, India.^18^ Another approach is to strengthen the involvement of other actors in the community health system in protecting CHW programmes and providing peer support to CHWs.^42^

Of the governance tasks listed in the framework, perhaps the best characterised and researched are those relating to the managerial building blocks of CHW programmes: namely, human resource, information, financing and supply chain systems.^43^ A key preoccupation across countries and regions is that of CHW motivation and retention, and the human resource practices that influence this – such as selection, scopes of practice, workload, community and health system support/supervision, remuneration, career advancement, and other incentives.^44-47^ These tasks often remain poorly addressed in national CHW programmes, and are the subject of a growing evidence base, which includes trials of supervision systems, and a growing interest in the role of technologies – especially mhealth – in community-based health systems.^48,49^

Less well described is the leadership of change and the processes of participation, feedback, learning and adaptation which underpin successful CHW programmes. In Zambia, the lack of participation of key actors in the design phases of the Community Health Assistant programme hampered its ownership at lower levels,^50^ including its subsequent integration into the district health system.^51^ In contrast, in Chhattisgarh State, one of the key mechanisms facilitating scaling up of the Mitanin (CHW) Programme was a system of “plural governance,” which privileged the voices of frontline actors and which established a culture of “constructive critique.”^18^

In order to satisfy demands from external or internal constituencies, CHW policies and programmes may be introduced without real political, institutional or budgetary backing. Although they may not control the politics of health sector decision making, health system actors can shape political commitment to CHW programmes. They can identify and make of use windows of opportunity to advocate for the establishment of a CHW programme, as occurred in Chhattisgarh. In Niger, implementation of reforms to the community-based sector were accelerated when they became aligned with broader political objectives.^52^ Positive evaluations of the Brazilian Family Health Programme^53^ and the Ethiopian Health Extension Worker Programme^54^ have raised their international profile and legitimacy. In recent years, Investment Cases have been developed as a methodology for drawing political attention to the value of CHW Programmes.^35^

## Limitations

In generating lessons for the development and leadership of national CHW programmes, the framework assumes that the ownership, oversight and ultimate regulatory control of national CHW programmes lies with public health systems. This is the trend for large-scale national programmes, certainly in Africa (of which the programmes in Ethiopia, Rwanda and Malawi are examples). It further envisions the presence of willing and politically astute governors acting in the public interest at the helm of CHW programmes, which may not be the case. The analysis thus has less relevance for contexts where the state is a weak player, by choice or not, in shaping CHW initiatives. However, in such instances it is doubtful whether a national CHW programme would be feasible although specific tasks/purposes may be relevant for thinking about more localised programmes. Unitary systems, with fewer negotiated interfaces and lower levels of sub-national autonomy, may require a less complex set of tasks and capacities. In other settings, the focal point of CHW programme governance may lie with other institutions (political, religious, non-governmental) within the community health system. For example, in Thailand, a very large community-based sector relies almost entirely on civil society voluntarism, with a strong link to faith-based organisations.^55^ Finally, a limitation of the framework it that it was developed on the basis of only 1 country’s experiences.

## Conclusion

Building from an empirical base in 1 middle-income country setting and drawing on the broader literature on health sector governance and CHW programmes in other countries, this paper has sought to systematise the governance function in CHW programmes, in order to shed light on how best to support and strengthen this function at scale.

The multi-level framework proposed serves as a heuristic device illustrating the range of roles and competencies required in practice and their distributed nature. It can be used as a tool to analyse and assess the governance of CHW programmes across levels of the system, and to design implementation strategies. It can also provide the basis for training and capacity development in the governance and leadership of CHW programmes at scale.

Further steps in the development of the framework would include: testing its validity in other contexts, and its value as a tool for planning, mapping and prospectively monitoring the governance and leadership of CHW programmes; and elaborating its components into curricula for continuing education and work-based learning.

## Acknowledgements

The author holds a Research Chair funded by the South African Research Chairs initiative of the Department of Science and Technology and National Research Foundation of South Africa (grant No. 98918). Any opinion, finding and conclusion or recommendation expressed in this material is that of the author(s) and the NRF does not accept any liability in this regard.

The inputs of my colleagues Uta Lehmann, Lucy Gilson, Di McIntyre, and the anonymous reviewers are gratefully acknowledged.

## Ethical issues

No primary data collection was conducted for this paper, which is based on a synthesis of the literature and previous analyses published by the author. Additional ethics clearance was thus not obtained.

## Competing interests

Author declares that she has no competing interests.

## Author’s contribution

HS is the single author of the paper.

## Key messages

**Implications for policy makers**

- Policy-makers are increasingly being asked to consider, develop or strengthen community health worker (CHW) programmes as part of their mandates.
- Long-standing experience suggests that national CHW programmes are neither cheap nor simple, yet there is relatively little guidance on how best to design and implement such programmes so that they sustainably achieve their goals.
- The aim of the framework presented in this paper is to systematise and organise the tasks of CHW programme governance as they present themselves to the stewards of these programmes, most often public sector managers.
- In doing so it seeks to provide a better appreciation of CHW programme governance functions across levels of the health system.

**Implications for public**

Community health workers (CHWs) are non-professional health cadres who are typically selected by local communities to provide limited health functions, mobilise communities around their health needs, and act as a bridge with the formal health system. They include the Behvarz in Iran, Health Extension Worker in Ethiopia, and Accredited Social Health Activist in India. Managing national CHW programmes is not simple as CHWs straddle two worlds (community and health system), often fall outside of formal public sector employment, and require particular kinds of support and supervision. Based on research in South Africa, this paper proposes a framework for the oversight and guidance of national CHW programmes, a function referred to as governance. It seeks to contribute to thinking on how to strengthen these programmes so that they make a real impact on the availability, affordability and acceptability of health services, and ultimately, the health of citizens.
